# Acoustic radiation force impulse imaging: normal values of spleen stiffness in healthy children

**DOI:** 10.1007/s00247-021-05079-8

**Published:** 2021-05-13

**Authors:** Sylviane Hanquinet, Céline Habre, Méryle Laurent, Mehrak Anooshiravani, Seema Toso

**Affiliations:** Department of Pediatric Radiology, University Children’s Hospital Geneva, 6 rue Willy Donzé, CH 1211 Genéve 14, Suisse

**Keywords:** Acoustic radiation force impulse, Children, Normal values, Shear wave elastography, Spleen, Stiffness, Ultrasound

## Abstract

**Background:**

Acoustic radiation force impulse (ARFI) imaging is a noninvasive ultrasound elastography technique for evaluating tissue stiffness. The association of liver and spleen stiffness provides additional information in the assessment of portal hypertension. The technique and normal values of spleen stiffness by point shear wave elastography (p-SWE) in pediatrics have not been well documented.

**Objective:**

Our aim is to describe the feasibility and normal ARFI elastography values in the spleen for healthy children and to compare measurements in two different probe positions (the axial and sagittal planes).

**Materials and methods:**

Spleen p-SWE using ARFI values were measured with a 6C1 probe in 102 healthy children (age range: 8 weeks to 17 years) divided into four age groups. An average of nine (standard deviation: two) spleen stiffness measurements were taken during free breathing in each plane (axial and sagittal). The impact of age and measurement plane in the spleen was analyzed using multivariate models.

**Results:**

There was no significant difference in spleen stiffness values taken at different ages, with an average of the medians of 2.43±0.31 m/s. There was no significant difference based on probe orientation: sagittal plane (median: 2.46±0.29 m/s) and axial plane (median: 2.43±0.32 m/s) with Student’s *t*-test *P*=0.18. The mean depth of measurement varied between 2.3 cm and 3.7 cm, according to age.

**Conclusion:**

Normal spleen stiffness values using ARFI imaging in children do not vary with age and correspond to a median of 2.43 m/s. No significant difference was found when using different probe positions.

## Introduction

In recent years, ultrasound (US) elastography has been developed in children to evaluate liver stiffness and quantify fibrosis. This rapid, noninvasive technique is of clinical interest in the management of children with chronic liver disease and/or liver transplants who are at risk of developing liver fibrosis and cirrhosis [1]. Recently, interest has developed in spleen elastography as a complementary measure to evaluate early signs of portal hypertension and secondary vascular complications and to predict or grade esophageal varices in adults and children [2, 3]. Indeed, splenomegaly secondary to congestion is a direct consequence of increased portal pressure in chronic liver disease and may result in increased splenic stiffness [4, 5].

Normal spleen stiffness values have not yet been standardized in the literature with acoustic radiation force impulse (ARFI) elastography, a quantitative point shear wave elastography (p-SWE), providing values in m/s.

Few pediatric publications exist with small series of patients and measurements performed on different US machines, a factor known to provide variability among values [6]. Only one study with 60 children was performed for ARFI spleen stiffness evaluation in healthy children [7]. That study established standard p-SWE values using two different probes, 9 MHz and 4 MHz, but only using a sagittal plane. We propose evaluating splenic stiffness in healthy children using a curved 6C1 HD MHz transducer. In comparison with the old probes (9 MHz and 4 MHz), the wait time between each measurement is clearly shortened and the study is fast and well tolerated by children.

Due to the anatomical and physiological differences related to pediatric populations, it is essential to establish a normal range of spleen stiffness measurements in children before analyzing patients with chronic liver disease. We also studied the technical feasibility of two acquisition techniques: intercostal access for the sagittal plane versus subcostal access for the axial plane.

We hypothesized that measurements may be influenced by patient age and also by the probe orientation because of the movement artefacts [8]. In this paper, we propose normal values of spleen stiffness using ARFI in a large pediatric population according to age distribution and probe orientation.

## Materials and methods

This prospective study, performed October 2019 to June 2020, was approved by the ethics committee of our hospital. According to the criteria for consent established by the ethics committee, written consent was obtained for each examination from either the patients and/or their parents.

We performed ARFI measurements in 102 presumed healthy children (age range: 8 weeks to 17 years). These children had undergone an abdominal US but did not have liver or spleen pathologies. The indications for US were multiple: kidneys (dilated renal cavities, malformation), musculoskeletal (hip dislocation, limping), genital (precocious puberty, malformations). For the majority of the children, the US indication did not require fasting. Children (or their guardians) were asked if the child was taking any medication. Inclusion criteria were absence of known liver/spleen disease based on history and normal standard abdominal US with normal value of liver elastography. Liver stiffness values were taken as a control to confirm the normality of the liver according to the standards in the literature [9, 10]. Splenic length was also measured and referred to age-based normal charts [11].

The ARFI elastography module is installed on a conventional US machine (Acuson Ultrasound System, S3000 platform; Siemens Healthineers, Erlangen, Germany). The p-SWE is based on sending a short, high-intensity, ultrasonic wave to a region of interest (ROI) in the target tissue. We obtained stiffness values expressed in m/s, which increase with the stiffness of the examined tissue. The technique is available with one recent probe, a curved 6C1 HD MHz transducer on the S3000 platform. This probe provides an ROI for elastography of 5×4 mm.

Children were in the supine position, and measurements were obtained with free breathing at the end of exhalation to increase technique homogeneity.

Each patient was examined by one of three senior pediatric radiologists (S.T., with 6 years of experience, M.A., with 12 years of experience, S.H., with 12 years of experience), experienced with routine liver ARFI elastography.

Five stiffness measurements were recorded in one region of the liver (right lobe, avoiding a visible vessel or bile duct by an intercostal or subcostal approach) as previously described by the authors [10].

**Fig. 1:**
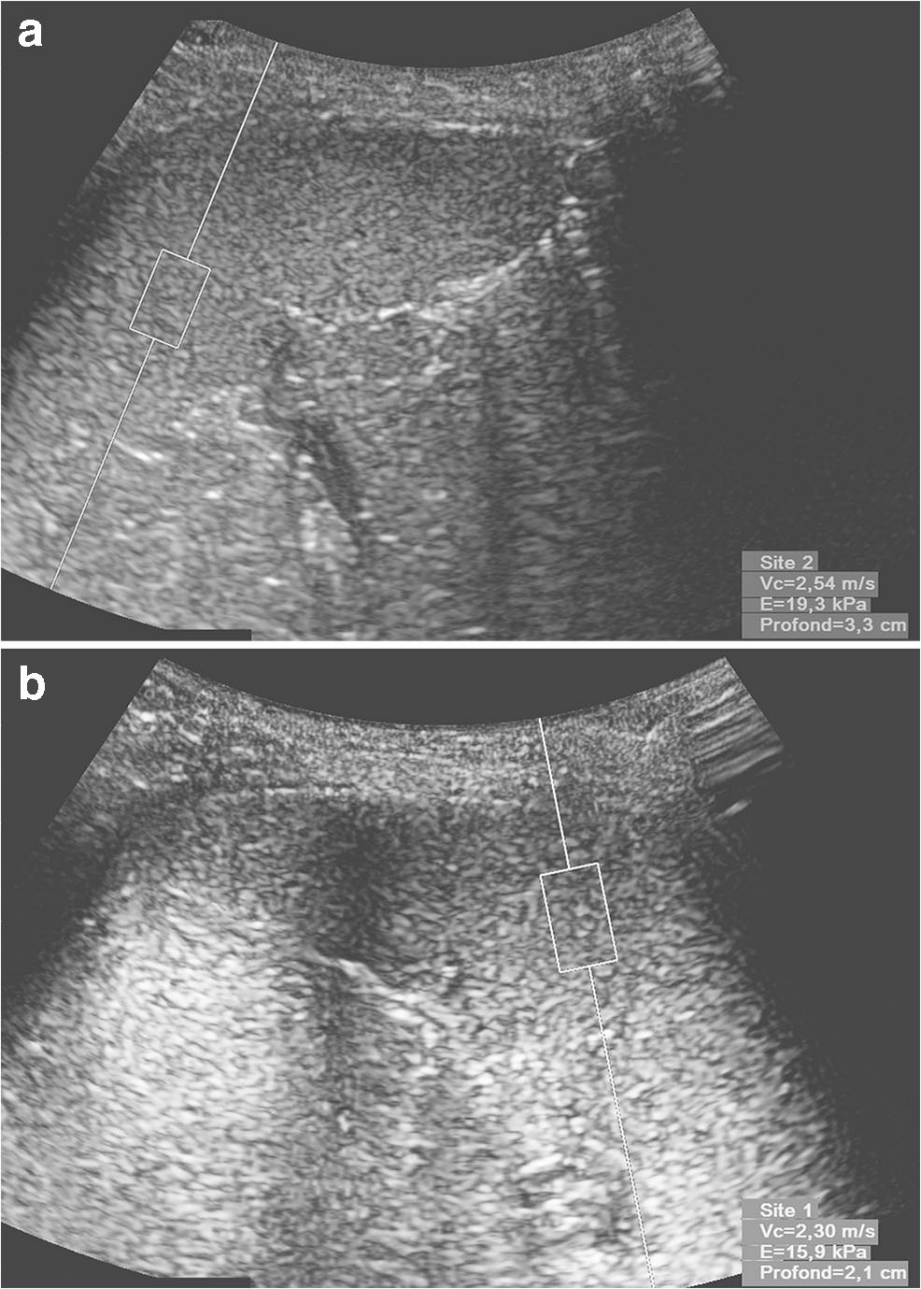
Acoustic radiation force impulse (ARFI) imaging measurements in a healthy 6-year-old boy using a 6C1 probe. **a** In the sagittal plane, shear wave velocity is 2.54 m/s at a depth of 3.3 cm. **b** In the axial plane, shear wave velocity is 2.30 m/s at a depth of 2.1 cm

Measurements in the spleen were taken in two orthogonal planes, positioning the probe in an axial plane and in a sagittal plane in order to best access the splenic parenchyma, away from the hilum, by subcostal access for the axial plane and intercostal positioning for the sagittal plane (Fig. 1). For each child, we tried to carry out 10 measurements per plane in the spleen.

Values indicated as “XXX” were considered invalid and were immediately eliminated. Valid ARFI values and measurement depths were recorded by the machine. The software calculated the mean, median and interquartile range to median (IQR/M) ARFI values. The quality criteria was defined as an IQR/M value ratio <30% [12, 13].

Descriptive statistics (mean, range, standard deviation [SD]) were used to present patient demographics, liver ARFI values and spleen ARFI values. An analysis of variance (ANOVA) test was used to determine whether significant differences in liver and in spleen (axial and sagittal) ARFI values existed between age groups. A paired Student’s *t*-test was used to test for differences between axial and sagittal spleen ARFI values. A Pearson’s test was used to assess linear correlation as function of age. All analyses were done using Python’s (Python Software Foundation, Beaverton, OR) SciPy open source library.

## Results

### Baseline characteristics (Table 1)

Of the 105 eligible children, we excluded three patients for whom valid measurements in the liver and spleen were not available due to poor compliance. Of the remaining 102 patients, the mean age was 6 years (range: 8 weeks to 17 years, SD: 5.1 years) with the majority being male (67%). The mean body mass index (BMI) was 16.9 kg/m^2^ (SD: 3.0 kg/m^2^). The population was divided into four age groups with a balanced number per group — group 1 (*n*=25): 0 to <1 year, group 2 (*n*=25): 1 to <5 years, group 3 (*n*=25): 5 to <10 years and group 4 (*n*=25): 10 to 17 years.

**Table 1:** Demographic data of the population by age group

	0–1 year *n*=25	1–5 years *n*=25	5–10 years *n*=25	10–17 years *n*=27
Age in years, mean±SD	0.4±0.3	2±1	7±1	13±2
Gender (F/M)	8/17	5/19	9/18	10/16
BMI (kg/m^2^), mean±SD	16.5±2.8	15.9±2.5	16±2.3	19.3±3.1
Spleen length (mm), mean±SD	51±7	70±9	86±9	101±12

*BMI* body mass index, *F* female, *M* male, *SD* standard deviation

The splenic lengths in the different age groups were as follows: <1 year: 51 mm (range: 40–68 mm, SD: 7 mm), 1 to <5 years: 70 mm (range: 54–89 mm, SD: 9 mm), 5 to <10 years: 86 mm (range: 60–105 mm, SD: 9 mm) and 10 to <17: 101 mm (range: 74–130 mm, SD: 12 mm).

All patients had normal hepatic stiffness values for their respective age groups as compared to relevant values in the literature [10]. The average median value in the liver was 1.03±0.15 m/s (IQR/M: 0.19±0.09 m/s).

### Splenic ARFI measurements

A total of 1,697 splenic stiffness measurements were retained for statistical analysis. For splenic stiffness, an average of nine (SD: two) measurements in the axial and sagittal planes was performed, after removing invalid measurements. Mean measurement depth for splenic ARFI varied according to age — <1 year: 2.3 cm (range: 1.5–3.7 cm, SD: 0.4 cm), 1 to <5 years: 2.7 cm (range: 2.0–3.9 cm, SD: 0.4 cm), 5 to <10 years: 3.1 cm (range: 2.0–5.5 cm, SD: 0.7 cm) and 10 to <17 years: 3.7 cm (range: 2.4–6.3 cm, SD: 0.8 cm).

**Fig. 2:**
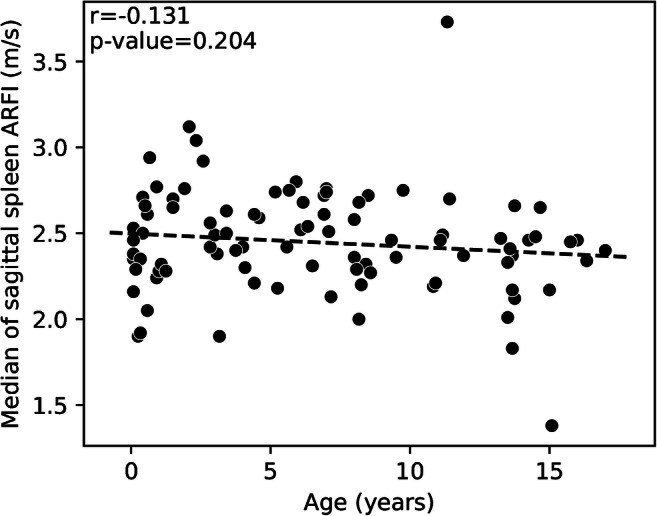
A scatterplot with a regression line of the relationship between age (years) along the horizontal axis and median sagittal spleen shear wave velocities acoustic radiation force impulse (ARFI) (m/s) along the vertical axis (*P*-value=0.20, r=−0.131)

**Fig. 3:**
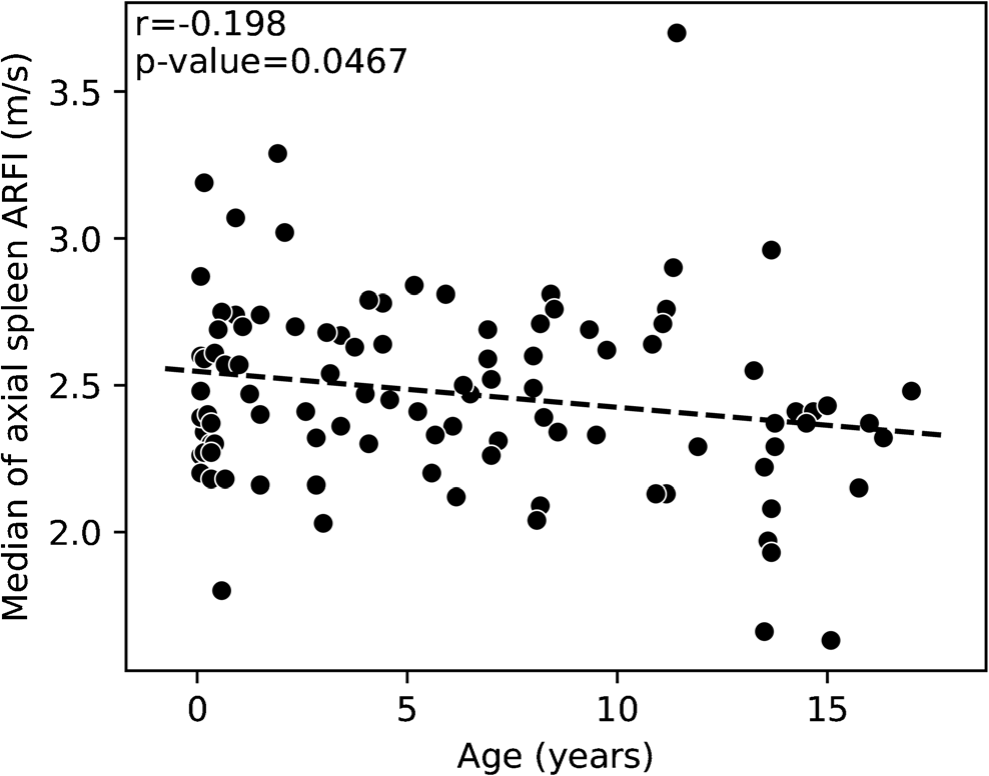
A scatterplot with a regression line of the relationship between age (years) along the horizontal axis and median of axial spleen shear wave velocities acoustic radiation force impulse (ARFI) (m/s) along the vertical axis (*P*-value=0.047, r=−0.198)

**Fig. 4:**
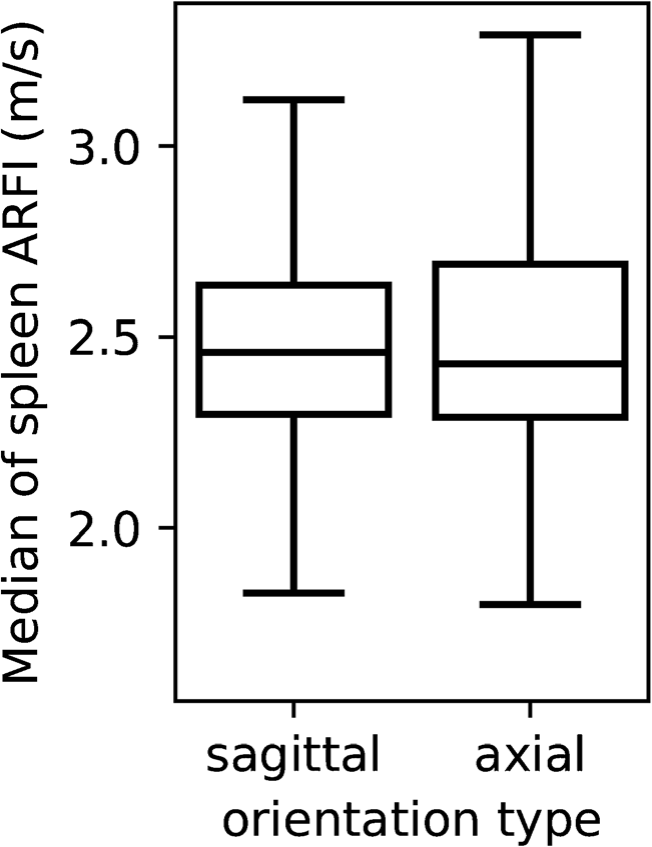
Box plots of median spleen elastography values between probe positions, axial and sagittal, in all populations. The line in the middle of the box is the median, the bottom and top of the box are the 25th and 75th percentiles. *ARFI* acoustic radiation force impulse

The median spleen stiffness was 2.43±0.31 m/s for the entire cohort. Spleen stiffness values by age and plane are available in Figs. 2 and 3. The average median value of spleen elastography for all children was 2.46±0.29 m/s in the sagittal plane and 2.43±0.32 m/s in the axial plane with Student’s *t*-test *P*-value=0.18 (Fig. 4).

The spleen values (median, range and SD) according to group age are as follows: in the sagittal plane, group 1: 2.44 (2.10–2.78, SD: 0.2), group 2: 2.50 (2.00–3.01, SD: 0.23), group 3: 2.50 (2.09–2.98, SD: 0.21) and group 4: 2.40 (1.48–3.61, SD: 0.38); and in the axial plane, group 1: 2.49 (1.93–3.28, SD: 0.28), group 2: 2.54 (2.10–3.16, SD: 0.24), group 3: 2.52 (2.02–2.88, SD: 0.22) and group 4: 2.42 (1.75–3.72, SD: 0.41).

**Fig. 5:**
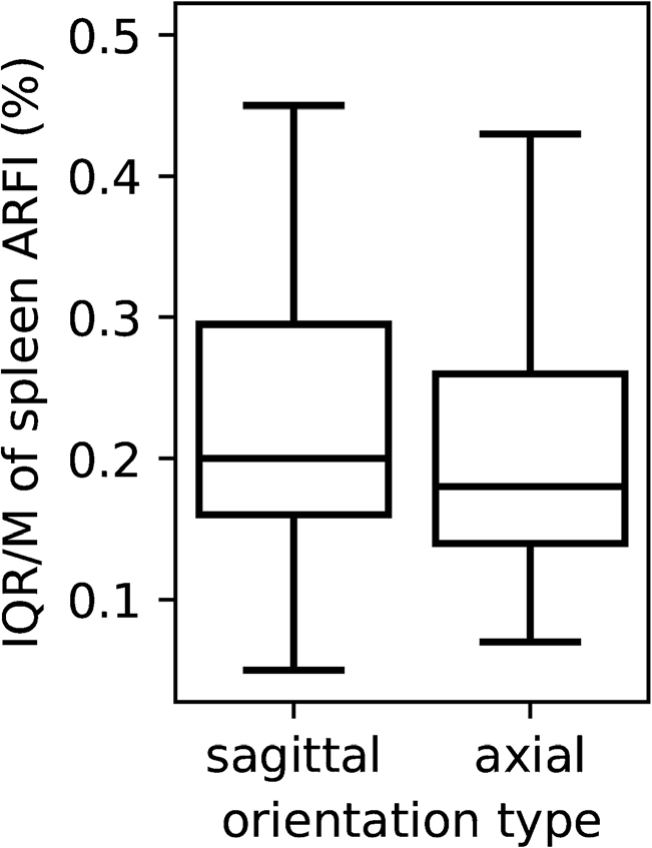
Box plots of interquartile range/median (IQR/M) of spleen elastography values between both probe positions, sagittal and axial. *ARFI* acoustic radiation force impulse

The values of IQR/M by age in the axial plane are as follows: group 1: 24%, group 2: 18%, group 3: 19% and group 4: 15%. In the sagittal plane, values of IQR/M by age are as follows: group 1: 29%, group 2: 22%, group 3: 17% and group 4: 19% We observed that the IQR/M in group 1 was higher compared to the other age groups (Mann-Whitney *U* test: *P*=0.000005) and also in the sagittal plane versus axial plane with Student’s *t*-test *P*=0.053 (Fig. 5).

## Discussion

The use of liver elastography by US is a well-known and standardized procedure in both adults and children. Hepatic elastography has become an indispensable complementary tool to basic abdominal US in all children with chronic liver disease to detect fibrosis. During the past decade, clinicians have shown increased interest in liver stiffness assessment to help manage patients with hepatic disease.

In recent years, some pediatric studies have analyzed the added value of spleen elastography to liver elastography for these patients. It appears that an increase in spleen stiffness might provide earlier indirect signs of portal hypertension than liver stiffness measurements [2, 3, 14, 15]. Portal hypertension manifests as venous congestion of the spleen and development of esophageal varices. For example, in the pediatric study by Tomita et al. [16], the cutoff value for splenic stiffness measurements was determined as 2.93 m/s, with 100% sensitivity and 78.9% specificity, but this study did not mention any normal values.

As stiffness measurements vary among manufacturers, each system must have its own reference values [17]. After using ARFI liver elastography with quantitative p-SWE over the last 10 years, we have found it is necessary to standardize spleen elastography techniques and to establish age-specific normal values for a large pediatric population, using more recent material, with the Siemens Acuson System with S3000 platform.

Some studies concerning splenic stiffness in children with chronic liver disease provide normal values in small control groups, but these were obtained from US machines from various manufacturers, thus compromising any reliable comparison of the values [6, 18]. Splenic stiffness measurements from these manufacturers were either expressed in kilopascal (kPa), e.g., with a median of 17.85±1.3 kPa on 10 children ages 4 to 14 years [5], or expressed both in kPa and m/s, e.g., with a median of 16.8 kPa (1.6–22.8 kPa) and 2.4 m/s (2.0–2.7 m/s) in 37 patients (0.5–18.5 years) [19].

Only two previous pediatric studies have reported normal values for splenic stiffness by ARFI and have described the technique in children on the same manufacturer’s US equipment. The first, performed by Canas et al. [7], reported normal values of spleen elastography by ARFI with two types of probes, one convex 4C1 and one linear 9L4. Sixty-eight children divided into 4 groups were recruited for the study and the mean shear wave velocities were 2.17 m/s (SD: 0.35 m/s) with the 4C1 probe and 2.15 m/s (SD: 0.23 m/s) with the 9L4 probe. Five measurements were obtained with vertical orientation to the spleen during free breathing. In view of their results, the authors divided the children into only two age groups [7]. In the second study, performed by Lee et al. [20], two probes were also used (9L4 and 4C1) and the mean value was 2.25±0.03 m/s. Their cohort was very large, including 200 children divided into 3 groups, but none <1 year of age. Only three measurements were made in the spleen and the probe orientation was not specified [20]. Although both studies provided necessary contributions to this literature, they both used 4C1 and 9L4 probes with a previous generation US machine (Acuson S2000).

Our splenic elastography results are slightly higher than those published by Canas et al. [7] and Lee et al. [20]. Compared to these studies that report normal values using ARFI, our study reports on a more homogenous age distribution between the four age groups and also includes very young children. Furthermore, from a statistical point of view, we performed more measurements per child (on average nine instead of five or three in other studies) and this may improve the final median values [7, 20]. On the other hand, our measurements were taken at end-expiration for the entire cohort (to increase homogeneity of the technique) because of the difficulty in having some children, often the younger ones, collaborate with breath-hold during the examination [21]. In our study, the average depth of the ROIs was 2.3–3.9 cm depending on age, contrary to others in the literature (range of depth 1.1–7.4 cm with the 4C1 probe [7] and 1.1–4.0 cm with the 9L4 probe [20]).

Furthermore, we have observed more variability (IQR/M) for measurements in children <1 year old probably due to movement artefacts and difficult to access/smaller spleens. We hypothesized that there would be less variability in measurements in the axial plane because we were able to better stabilize our probe and perhaps better control movement artefacts. However, our results showed no significant difference in spleen stiffness based on plane orientation.

This study has several limitations. Based on the number of cases, our sub-analysis was grouped by age. We were unable to do a sex-based sub-analysis because there were not enough cases per age group and sex available. We have not established intra−/interobserver variability, but the measurements were performed by senior pediatric radiologists with many years of experience in the ARFI technique [1, 10, 22, 23]. Another limitation of this study concerns the inclusion criteria of presumably healthy children. Patients who were cited as otherwise healthy with no known medical conditions were presumed healthy and included in the study. Laboratory confirmation was not performed as this was considered too invasive. To manage this limitation, we ensured a normal liver US, liver stiffness measurements and normal spleen length as part of our inclusion criteria. Fasting is recommended for liver elastography and we are aware of this limitation in our study. These was no strict fasting since it was not necessary for the US indications. Despite this limitation, our liver stiffness values were within normal limits.

It is interesting to note that we observed higher normal spleen stiffness values compared to normal liver stiffness values in our population. This has not yet been reported in the literature in the same pediatric population using the same technique. Early detection of portal hypertension might benefit from the addition of spleen elastography to liver elastography in pediatric chronic liver disease to obtain additional information, and further work is needed in this field. However, having baseline normal splenic values in children is the first step in this process.

## Conclusion

The normal spleen p-SWE using ARFI values are on average 2.4 m/s and therefore higher than those of the liver, which are around 1 m/s. For chronic liver disease in children, US liver elastography is part of the basic conventional abdominal US examination and the addition of spleen stiffness measurements may provide additional information in the evaluation of portal hypertension. This technique is feasible in children, regardless of age. We recommend positioning the US probe in an axial rather than sagittal plane to obtain values that are less affected by respiratory movements, especially in young children.

## References

[1] Hanquinet S, Rougemont AL, Courvoisier D (2013). Acoustic radiation force impulse (ARFI) elastography for the noninvasive diagnosis of liver fibrosis in children. Pediatr Radiol.

[2] Cassinotto C, Charrie A, Mouries A (2015). Liver and spleen elastography using supersonic shear imaging for the non-invasive diagnosis of cirrhosis severity and oesophageal varices. Dig Liver Dis.

[3] Sintusek P, Siriporn N, Punpanich D (2019). Spleen and liver stiffness to detect esophageal varices in children with biliary atresia. J Pediatr Gastroenterol Nutr.

[4] Tomita H, Ohkuma K, Masugi Y (2016). Diagnosing native liver fibrosis and esophageal varices using liver and spleen stiffness measurements in biliary atresia: a pilot study. Pediatr Radiol.

[5] Yuldashev RZ, Aliev MM, Shokhaydarov SI (2020). Spleen stiffness measurement as a non-invasive test to evaluate and monitor portal hypertension in children with extrahepatic portal vein obstruction. Pediatr Surg Int.

[6] Dillman JR, Chen S, Davenport MS (2015). Superficial ultrasound shear wave speed measurements in soft and hard elasticity phantoms: repeatability and reproducibility using two ultrasound systems. Pediatr Radiol.

[7] Canas T, Fontanilla T, Miralles M (2015). Normal values of spleen stiffness in healthy children assessed by acoustic radiation force impulse imaging (ARFI): comparison between two ultrasound transducers. Pediatr Radiol.

[8] Halabi SS (2021). Deep learning augments liver stiffness classification in children. Pediatr Radiol.

[9] Fontanilla T, Canas T, Macia A (2014). Normal values of liver shear wave velocity in healthy children assessed by acoustic radiation force impulse imaging using a convex probe and a linear probe. Ultrasound Med Biol.

[10] Hanquinet S, Courvoisier D, Kanavaki A (2013). Acoustic radiation force impulse imaging-normal values of liver stiffness in healthy children. Pediatr Radiol.

[11] Megremis SD, Vlachonikolis IG, Tsilimigaki AM (2004). Spleen length in childhood with US: normal values based on age, sex, and somatometric parameters. Radiology.

[12] Barr RG, Wilson SR, Rubens D (2020). Update to the Society of Radiologists in Ultrasound liver elastography consensus statement. Radiology.

[13] Northern NA, Dillman JR, Trout AT (2019). Frequency of technical success of two-dimensional ultrasound shear wave elastography in a large pediatric and young adult cohort: a clinical effectiveness study. Pediatr Radiol.

[14] Canas T, Macia A, Munoz-Codoceo RA (2015). Hepatic and splenic acoustic radiation force impulse shear wave velocity elastography in children with liver disease associated with cystic fibrosis. Biomed Res Int.

[15] Goldschmidt I, Brauch C, Poynard T (2014). Spleen stiffness measurement by transient elastography to diagnose portal hypertension in children. J Pediatr Gastroenterol Nutr.

[16] Tomita H, Fuchimoto Y, Ohkuma K (2015). Spleen stiffness measurements by acoustic radiation force impulse imaging after living donor liver transplantation in children: a potential quantitative index for venous complications. Pediatr Radiol.

[17] Gilligan LA, Trout AT, Bennett P, Dillman JR (2020). Repeatability and agreement of shear wave speed measurements in phantoms and human livers across 6 ultrasound 2-dimensional shear wave elastography systems. Investig Radiol.

[18] Alrashed AI, Alfuraih AM (2020). Reproducibility of shear wave elastography among operators, machines, and probes in an elasticity phantom. Ultrasonography.

[19] Caliskan E, Atay G, Kara M (2019). Comparative evaluation of liver, spleen, and kidney stiffness in HIV-monoinfected pediatric patients via shear wave elastography. Turk J Med Sci.

[20] Lee M-J, Kim M-J, Han KH, Yoon CS (2013). Age-related changes in liver, kidney, and spleen stiffness in healthy children measured with acoustic radiation force impulse imaging. Eur J Radiol.

[21] Karlas T, Lindner F, Tröltzsch M, Keim V (2014). Assessment of spleen stiffness using acoustic radiation force impulse imaging (ARFI): definition of examination standards and impact of breathing maneuvers. Ultraschall Med.

[22] Hanquinet S, Courvoisier DS, Rougemont AL (2016). Acoustic radiation force impulse sonography in assessing children with biliary atresia for liver transplantation. Pediatr Radiol.

[23] Hanquinet S, Courvoisier DS, Rougemont AL (2015). Contribution of acoustic radiation force impulse (ARFI) elastography to the ultrasound diagnosis of biliary atresia. Pediatr Radiol.

